# Supplementary material for: Potential molecular mechanisms of ETV6-RUNX1-positive B progenitor cell cluster in acute lymphoblastic leukemia revealed by single-cell RNA sequencing
Source: PeerJ. 2024 Nov 1;12:e18445. doi: 10.7717/peerj.18445 (PMC11533907; doi:10.7717/peerj.18445)
Supplement: Supplemental Information 2 [file peerj-12-18445-s002.docx]

| Changes needed |
| --- |
| **Action these changes and resubmit** |
| Thank you for resubmitting and for your patience. We are working through submissions in the order they are submitted. Please address them all before resubmitting.  **1. Permissions**  Figure 1 appear to have maps that may be copyrighted.  In a [Confidential Note to PeerJ Staff](https://peerj.com/manuscripts/104242/declarations/#other), please confirm that:   - Your figure(s) do not contain copyrighted material. Or: - Your figures contain copyrighted material and the copyright holder has completed our photo/video permission form found [**here**](https://peerj.com/about/author-instructions/#figure-referencing). Authors must obtain the necessary permission to reuse third-party material in the article. **You must supply a copy of the completed form if asked.**   Reply: We confirm that the figure 1 doesn’t contain copyrighted material. Thank you.  **2. Figure Accessibility**  Please adjust the red/green colors used on your figure 4 to make them accessible to those with color blindness OR add labels so the figures aren't solely dependent on color to differentiate between the elements. Please review our [**color blindness guidelines for figures**](https://peerj.com/about/author-instructions/#figure-style). Note: Please do **not** replace the red/green colors with patterns in your figures.  Please provide replacement figures measuring minimum 900 pixels and maximum 3000 pixels on all sides, saved as PNG, EPS or vector PDF file format without excess white space around the images. Do not supply figures in Word processing files. Do not change any other contents of the figures. Files must be named with the figure numbers: "Figure 1.png."  Reply: We have revised the Figure 4. Thank you. |
